# Targeted Cancer Therapy with Gold–Iron Oxide Nanourchins: Inducing Oxidative Stress, Paraptosis, and Sensitizing Tumor Cells to Cisplatin

**DOI:** 10.3390/antiox14040422

**Published:** 2025-03-31

**Authors:** Jessica Ruzzolini, Cecilia Anceschi, Martin Albino, Elena Balica, Beatrice Muzzi, Claudio Sangregorio, Elena Frediani, Noemi Formica, Francesca Margheri, Anastasia Chillà, Gabriella Fibbi, Anna Laurenzana

**Affiliations:** 1 Department of Experimental and Clinical Biomedical Sciences “Mario Serio”, University of Florence, 50134 Florence, Italy; cecilia.anceschi@unifi.it (C.A.); elena.frediani@unifi.it (E.F.); noemi.formica@unifi.it (N.F.); francesca.margheri@unifi.it (F.M.); anastasia.chilla@unifi.it (A.C.); gabriella.fibbi@unifi.it (G.F.); 2 Institute of Chemistry of Organometallic Compounds—C.N.R., 50019 Florence, Italy; martin.albino@unifi.it (M.A.); beatrice.muzzi@unifi.it (B.M.); claudio.sangregorio@unifi.it (C.S.); 3 Department of Chemistry ‘Ugo Schiff’ & INSTM, University of Florence, 50019 Florence, Italy; elena.balica@unifi.it

**Keywords:** nanoparticles, lung cancer, oxidative stress, paraptosis, chemotherapy potentiation

## Abstract

Nanotechnology has revolutionized cancer therapy by enabling targeted drug delivery and overcoming limitations associated with conventional chemotherapy. In this study, we explored the anticancer potential of gold–iron oxide (Au-Fe_3_O_4_@PEG) nanourchins (NUs), a class of nanoparticles with unique shape, surface features, and plasmonic properties. We tested NUs on several cancer cell lines, including A375 (melanoma), MCF7 (breast), A549 (lung), and MIA PaCa-2 (pancreatic), and observed significant dose-dependent cytotoxicity, with A549 cells exhibiting the highest resistance. Our findings also demonstrate that NUs induce oxidative stress, disrupt mitochondrial function, and activate autophagic and paraptotic cell death pathways in A549 lung cancer cells. Additionally, we explored the potential of NUs to enhance the efficacy of platinum-based chemotherapy, specifically cisplatin, in A549. The results provide valuable insights into the therapeutic potential of NUs in the context of cancer treatment, particularly for overcoming drug resistance and enhancing the effectiveness of conventional chemotherapy.

## 1. Introduction

Cancer still represents one of the main causes of death in the world, necessitating continuous efforts to discover new therapeutic approaches, particularly for highly malignant tumors. Among these, lung cancer stands out due to its high metastatic potential and substantial global burden, accounting for approximately 11.6% of all diagnosed cancers [1,2]. This alarming prevalence makes lung cancer a critical focus for research into innovative treatments. Nearly 85% of lung cancers are classified as non-small-cell lung cancer (NSCLC), while the remaining part belongs to small-cell lung cancer (SCLC) [3]. Current treatments for lung cancer include surgery, chemotherapy, radiation, and targeted therapy alone or in combination with each other according to disease stage and type [4,5]. Despite these options, the first-line therapy for NSCLC remains chemotherapy, typically involving cisplatin and paclitaxel [6]. However, the effectiveness of chemotherapy is often hindered by the development of drug resistance and its cytotoxic effects on normal cells, highlighting the need for innovative therapeutic strategies. In recent decades, nanomedicine has emerged as a promising avenue in oncological research, driven by the unique properties of nanoparticles (NPs), such as their size, stability, and modifiable surfaces [7]. The application of NPs in cancer therapy offers several advantages, including targeted drug delivery to specific tissues or cells, enhanced drug stability and solubility, prolonged circulation times, and increased tumor accumulation [8]. Typically, nanoparticles are composed of a core, a shell layer, and a surface layer, with the latter often modified using polyethylene glycol (PEG) to evade immune clearance [9,10]. Among these, metal NPs are widely used for their exceptional optical, catalytic, and antimicrobial properties [11]; gold and silver ones especially show high stability and efficient capability to convert and manipulate energy and matter at the nanoscale [12]. Gold nanoparticles (AuNPs), in particular, show high anticancer activity; they can cause tumor damage by photothermal ablation, as a drug delivery system for drugs, but also in a mechanical way [13]. Kodiha M. et al. [13] summarized the impact of AuNPs on cancer cell survival, collecting evidence that this kind of NPs can enter the nucleus and mitochondria, impairing cellular functions and leading to cell death. Also, some authors found that AuNPs can exert their anticancer activity by targeting the endoplasmatic reticulum (ER), as demonstrated in B16F10 melanoma cells, Hela cells, Kupffer cells, and K562 cells [14,15,16,17,18]. Gold nanourchins are NPs with unique properties and surface features, showing advantages over gold nanoparticles [8,19,20]. In this study, we tested the effects of pegylated gold–iron oxide nanourchins (Au-Fe_3_O_4_@PEG Nus) on A549 lung cancer cells, and we demonstrated their efficacy in inducing oxidative stress, autophagy, and, finally, death through mitochondrial damage.

## 2. Materials and Methods

### 2.1. Cell Lines and Culture Conditions

Melanoma cell line A375M6 was isolated from a lung metastasis of SCID bg7bg mice i.v. injected with A375p obtained from American Type Culture Collection (ATCC, Rockville, MD, USA) and validated through STR profile (BMR Genomics, Padua, Italy) [21,22]. Breast cancer MCF7, pancreatic carcinoma MIA PaCa-2, and lung carcinoma A549 cells were purchased from ATCC, while the neonatal human dermal fibroblasts (NHDFs) were obtained from Lonza. A375M6, MCF7, MIA PaCa-2, and NHDF cells were cultivated in Dulbecco’s Modified Eagle Medium high glucose (DMEM 4500, EuroClone, Milan, Italy) supplemented with 10% fetal bovine serum (FBS, Boehringer Mannheim, Germany), at 37 °C in a humidified atmosphere containing 90% air and 5% CO_2_. A549 cells were cultured in RPMI-1640 supplemented with 10% FBS at 37 °C in a humidified atmosphere containing 90% air and 5% CO_2_.

Cells were harvested from subconfluent cultures by incubation with a trypsin–EDTA solution (EuroClone, Milan, Italy) and propagated every three days.

### 2.2. Nanourchins Synthesis

Au-Fe_3_O_4_@PEG Nus (Nus) were synthesized by using a seeded-mediated growth method starting from Au-Fe_3_O_4_ nano-dumbbells obtained by thermal decomposition of iron pentacarbonyl (Fe(CO)_5_) in the presence of preformed gold nanoseeds. Then, highly anisotropic Au-Fe_3_O_4_ NUs were synthesized by quickly adding Au-Fe_3_O_4_ dumbbells-like NPs to a pre-reduced Au+ growth solution containing tetra-chloroauric (III) acid trihydrate (HAuCl_4_·3H_2_O), CTAB, silver nitrate (AgNO_3_), and L-ascorbic acid. Finally, to enhance the colloidal stability and to confer biocompatibility to the system, Au-Fe_3_O_4_ NUs were functionalized with thiol-terminated polyethylene glycol (MeO-PEG5000-SH and HS-PEG5000-COOH).

### 2.3. MTT Assay

The 10 × 10^3^ cells/well were seeded on a 96-well plate in a completely fresh medium. One day later, cells were treated with the desired concentration of NUs and cisplatin, and after 72 h, the medium was removed, and cells were incubated for 1.5 h with completely fresh media without phenol red supplemented with 0.5 mg/mL MTT (3-(4,5-dimethyl-2 thiazolyl)-2,5-diphenyl-2H-tetrazolium bromide; Sigma Aldrich, Milan, Italy). Cells were then lysed in 100 mL of DMSO (SigmaAldrich, Milan, Italy). The blue formazan absorbance was automatically read at 595 nm using a spectrophotometric microplate reader (Biorad, Milan, Italy). The results were analyzed using the GraphPad Prism 10 software as described in [23]. Cell viability under each treatment was compared to untreated controls and calculated as 100% of viability.

### 2.4. Oil Red O Staining

Cellular monolayer was washed with phosphate-buffer saline (PBS), then fixed in formalin (10%) for 30 min at room temperature (RT). Formalin was discarded by washing cells twice with distilled water; then, cells were rinsed with isopropanol 60% for 5 min RT before proceeding with freshly prepared Oli Red O (SigmaAldrich, Milan, Italy) staining for 20 min RT. Finally, the excess stain was removed by washing cells with distilled water.

### 2.5. Cell Rox Staining for Oxidative Stress Detection

The 10 × 10^3^ cells/well were seeded in a compete medium in a 96-well plate for Luminometer (BioTek, Winooski, VT, USA). The next day, cells were treated with different doses of NUs and with 300 μM H_2_O_2_ for 24 h as a positive control for ROS and with N-acetyl-L-cystein (NAC) 3 mM as ROS inhibitor. NAC was administered for 2 h; then, the medium was removed and replaced with fresh containing NUs. After 24 h of treatment, culture media were removed, and cells were incubated for 30 min at 37 °C with CellROX^®^ Green Reagent (Thermofisher, Monza, Italy) at a final concentration of 5 μM. Then, cells were washed three times with PBS, and fluorescence intensity at 488 nm was measured using a Sinergy H1 plate reader (BioTek, Winooski, VT, USA).

### 2.6. Bodipy Staining for Lipidic Peroxidation Evaluation

The 30 × 10^3^ cells/well were seeded in an m-slide well with a high glass bottom (IBIDI, Gräfelfing, Germany). After 24 h of treatments, cells were incubated with BODIPY™ 581/591 C11 Lipid Peroxidation Sensor (Thermofisher, Monza, Italy) at the final concentration of 10 mM in complete medium for 1 h at 37 °C protected from light. Then, BODIPY was removed, and cells were washed three times with PBS. Cell nuclei were labeled with DAPI 1 µg/mL (Sigma-Aldrich, Schnelldorf, Germany) for 20 min RT. After washes in PBS, the reagent fluorescence emission peak shift from ~590 nm (red) to ~510 nm (green), indicating lipidic peroxidation, was detected using an inverted confocal microscope (Leica SP8 Confocal Microscope, Leica Microsystems, Wetzlar, Germany).

### 2.7. JC-1 Assay for Mitochondrial Membrane Potential Analysis

The 30 × 10^3^ cells/well were seeded in an m-slide well with a high glass bottom. After 3 h of NUs treatment, cells were labeled with a JC-1 probe (Thermofisher, Monza, Italy) at 10 μg/mL concentration in a complete medium for 30 min RT protected from light. Then, cells were washed three times with PBS, and cell nuclei were stained with DAPI 1 µg/mL for 20 min RT. Cells were washed twice with PBS; the orange-red fluorescence, referred to as polarized mitochondria, and the green one, referred to as depolarized mitochondria, were detected, respectively, at ~590 and ~525 using the inverted confocal microscope.

### 2.8. Seahorse Metabolic Flux Analyses

Seahorse analysis was performed as previously described in [24] using the Seahorse XF Mito Stress Test Kit (Agilent Technologies, Santa Clara, CA, USA) to assess the reliance of cells on oxidative metabolism. After 72 h of treatment with different doses of NUs, cells were counted, and 30 × 10^3^ cells/well were suspended in XF Assay Medium supplemented with 2 mM glutamine and seeded in XF96 Seahorse^®^ microplates (Seahorse Bioscience, Billerica, MA, USA) precoated with poly-D-lysine (ThermoFisher Scientific, Waltham, MA, USA). The plate was left to equilibrate in a CO_2_-free incubator before being transferred to the Seahorse XF96 analyzer. The pre-hydrated cartridge was filled with the indicated compounds and calibrated for 30 min in the Seahorse Analyzer. All the experiments were performed at 37 °C. Normalization of protein content was performed after each experiment. The Seahorse XF Report Generator automatically calculated the parameters from Wave data that were exported to Excel or GraphPad.

### 2.9. Western Blotting

Western Blotting was performed as described in [23,25]. The cells were lysed in RIPA lysis buffer (Merck Millipore, Vimodrone, Milan, Italy), and the proteins were separated using electrophoresis; equal amounts of protein in Laemmmli buffer were separated on Bolt^®^ Bis-Tris Plus gels 4–12% precast polyacrylamide gels (Life Technologies, Monza, Italy). Fractionated proteins were transferred from the gel to a PVDF nitrocellulose membrane using the iBlot 2 system (Life Technologies, Monza, Italy). Then, the membrane was probed at 4 °C overnight with primary antibodies diluted in a solution of 1:1 Odyssey blocking buffer/T-PBS buffer. The primary antibodies were rabbit anti-Alix (1:1000, Cell Signaling Technology, distributed by Euroclone, Milan, Italy), rabbit anti p62 (1:1000, Cell Signaling Technology), and rabbit anti-LC3a/b (1:1000, Cell Signaling Technology). After the incubation with the primary antibody, the membrane was washed in a T-PBS buffer and incubated for 1 h at RT with a goat anti-rabbit IgG Alexa Fluor 750 antibody or with a goat anti-mouse IgG Alexa Fluor 680 antibody (Invitrogen, Milan, Italy), and then visualized using an Odyssey Infrared Imaging System (LI-COR^®^ Bioscience, distributed by Carlo Erba, Milan, Italy). The rabbit anti-vinculin (1:1000, Cell Signaling Technology) was used to assess the equal amounts of protein loaded in each lane.

### 2.10. Autophagy Assessment Through CYTO-ID ENZO Detection Kit

The 50 × 10^3^ cells/well were seeded in a 12-well plate and, the next day were treated with different doses of NUs. After 24 h of treatment, the cell medium was removed, and cells were incubated with CYTO-ID^®^ Green Detection Reagent suspended in 1X Assay Buffer (Enzo, distributed by Euroclone, Milan, Italy) 1 mL/mL for 30 min 37 °C according to the manufacturer’s instructions. Then, cells were washed with 1X Assay Buffer, and green fluorescence was detached using Invitrogen EVOS M5000 Cell Imaging System (ThermoFisher Scientific, Waltham, MA, USA). Finally, for the green fluorescence quantification, cells were detached with accutase (Euroclone, Milan, Italy) and analyzed using flow cytometry (BD-FACS Canto, Becton, Dickinson & Company, Franklin Lakes, NJ, USA).

### 2.11. Cell Cycle Analysis

Cell cycle distribution across the G0/G1, S, and G2/M phases was analyzed as described in [21] using propidium iodide (PI) (SigmaAldrich, Milan, Italy) staining. Cells were centrifuged and stained with a solution of 50 μg/mL PI, 0.1% trisodium citrate, and 0.1% NP40 (or triton x-100) in the dark at 4 °C for 30 min. Then, red propidium-DNA fluorescence was analyzed by flow cytometry (BD-FACS Canto).

### 2.12. Invasion Assay Through Geltrex

Cell invasion was evaluated through a Geltrex (0.25 μg/mL) (ThermoFisher Scientific, Waltham, MA, USA) basement membrane matrix using Millicell cell culture Insert (24-well PCF 8.0 mm, Millipore, Billerica, MA, USA). After 72 h of NUs treatment, cells were counted, and 10 × 10^3^ cells were seeded in the upper compartment of cell culture in 200 mL of complete fresh medium. A total of 300 mL of complete fresh medium was also added to the lower compartment. After 6 h of incubation (37 °C, 5% CO_2_), the membranes were fixed overnight in ice-cold methanol (SigmaAldrich, Milan, Italy). Cells on the upper side of the filters were wiped off using cotton swabs, while cells on the lower side of the filters were stained with Diff Quick solutions and counted.

### 2.13. In Vitro Tube Formation Assay

After a 72 h treatment, 1.8 × 10^4^ cells were plated onto precoated GelTrex plates in fresh medium. Images were captured 18 h later using an optical microscope. Quantification of nodes, junctions, segments, and pieces was provided by the Angiogenesis Analyzer tool of ImageJ software https://imagej.net/ij/ (accessed on 27 March 2025) as described in [26].

### 2.14. Evaluation of Apoptosis Through Cytofluorimetric Annexin V/PI Double Staining

The 50 × 10^3^ cells/well were seeded in a 12-well plate and, the next day, were treated with NUs alone or in combination with cisplatin. Following 72 h of treatment, culture media were collected, and cells were detached using acutase. Then, cells were centrifuged and washed using the annexin binding buffer (10 mM Hepes pH 7.4; 140 mM NaCl; 25 mM CaCl_2_). Cells were stained in 100 mL of annexin binding buffer containing 3 mL of Annexin V APC-conjugated (ImmunoTools, Friesoythe, Germany) and 1 μL of 100 μg/mL PI working solution and incubated for 15 min RT. Before proceeding with flow cytometer analysis, 400 mL of annexin binding buffer was added to each sample. Ten thousand events were collected to establish viable, early apoptotic, late apoptotic, or necrotic cells.

### 2.15. Statistics

As described in [23], the results are obtained from at least three independent experiments and expressed as means ± SD. The GraphPad Prism program was used to perform multiple comparison tests as specified in each figure legend. The statistical significance was accepted at *p* < 0.05.

## 3. Results

### 3.1. NUs Efficacy on Different Tumor Cell Types

**Figure 1:**
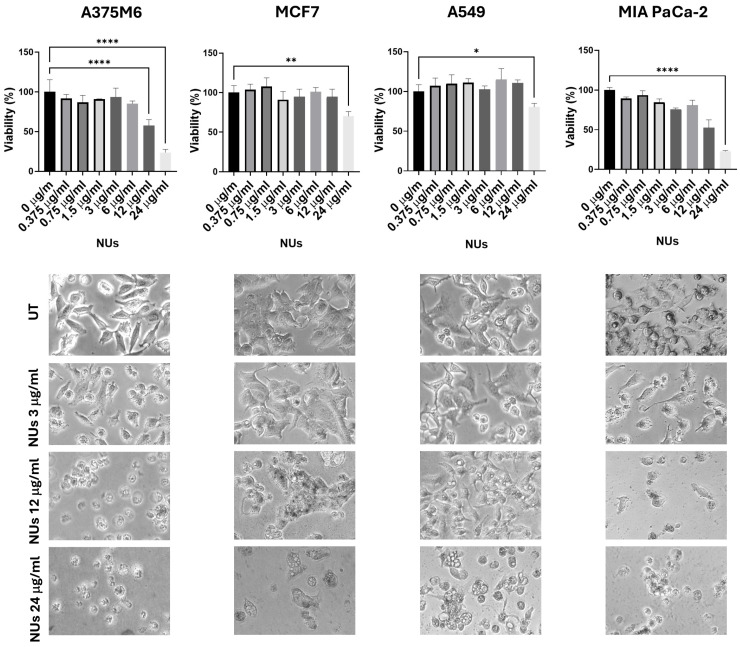
MTT assay of cells treated with different doses of NUs for 24 h and pictures of cells obtained with an inverted microscope at 20× magnification below. One-way analysis of variance (ANOVA), GraphPad Prism. * *p* < 0.05, ** *p* < 0.005, **** *p* < 0.0001.

Au-Fe_3_O_4_@PEG NUs were synthFesized by a colloidal seed-mediated approach. They comprise a small Fe_3_O_4_ nanosphere (~11 nm) encapsulated within a gold nano-urchin shell featuring highly elongated and irregular spiky tips (~131 nm). These nanoparticles were tested at increasing concentrations (0.375, 0.75, 1.5, 3, 6, 12, 24 μg/mL) on four different cell lines: A375M6 (melanoma), MCF7 (breast cancer), A549 (lung cancer), and MIA PaCa-2 (pancreatic cancer). The MTT assay results, presented in Figure 1, revealed that A549 cells exhibited significantly greater resistance to NUs across all tested concentrations compared to the other cell lines. Microscopic images of cells treated with 3, 12, and 24 μg/mL provided visual evidence of dose-dependent changes in cell morphology for A375, MCF7, and MIA PaCa-2 cells, which closely correlated with the observed decrease in cell viability. A375 and MIA PaCa-2 cells exhibited significant morphological changes at the highest concentration (24 μg/mL), including detachment from the culture plate, indicative of cell death. Additionally, A375M6 cells displayed cell blebbing even at lower concentrations, suggesting an early apoptotic response. In contrast, the only morphological change observed in A549 cells at the highest concentration, which was shared with MCF7 cells, was the appearance of vacuoles. These findings suggest that the morphological changes observed are tightly linked to the loss of cell viability induced by NU treatment. Moreover, the images demonstrate the accumulation of nanoparticles within the cells, as visualized by the presence of black dots. Given that A549 cells maintained their morphology and adhesion across all concentrations, consistent with their higher resistance and relatively unchanged viability in the MTT assay, we selected A549 cells as the focus for subsequent experiments to further investigate the role of NUs.

**Figure 2:**
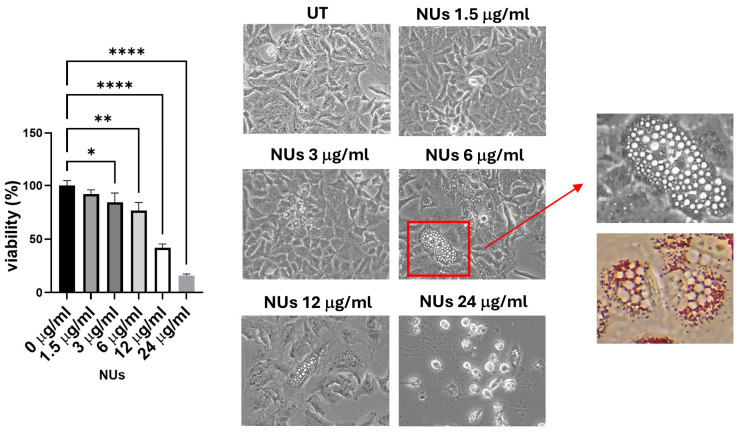
MTT assay and pictures of A549 cells obtained with an inverted microscope at 20× magnification after different doses of NUs for 72 h. Enlarged detail of cellular vacuolization after 72 h of 6 μγ/mL treatment in A549 cells before (above) and after Oil Red O staining (below). One-way ANOVA, GraphPad Prism. * *p* < 0.05, ** *p* < 0.005, **** *p* < 0.0001 MTT assay of A549 cells treated with different doses of NUs for 72 h. One-way analysis of variance (ANOVA), GraphPad Prism. * *p* < 0.05, ** *p* < 0.005.

Treatment with NUs for 72 h resulted in a significant reduction in A549 cell viability, with effects observed at low concentrations (3 μg/mL) and becoming more pronounced at 24 μg/mL, where cell viability dropped to approximately 15% (Figure 2). Additionally, NUs-treated cells displayed the formation of cytoplasmic vacuoles resembling lipid droplets. Given the findings of Kim et al. [27], which suggested that A549 cells could differentiate into adipocytes, forming adiposome-like vesicles positive for Oil Red O staining, we evaluated whether NUs treatment induced a similar adipocytic differentiation. However, Oil Red O staining yielded negative results, ruling out the lipidic nature of the observed vacuoles (Figure 2).

### 3.2. Pro-Oxidant Activity of NUs on A549 Cells

**Figure 3:**
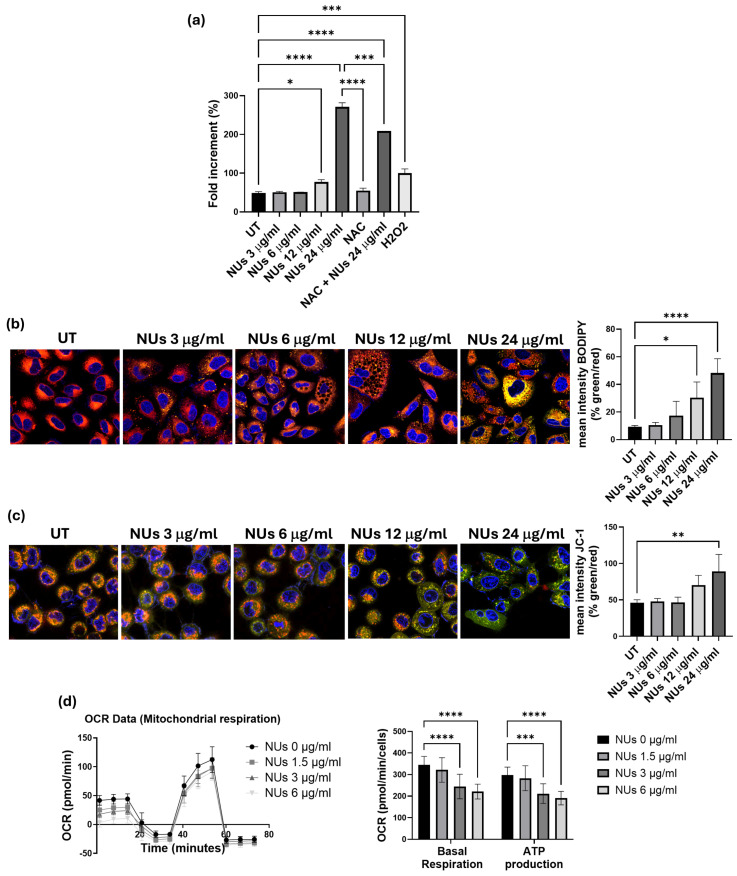
(**a**) CellROX fluorescence intensity after 24 h NUs treatment measured using a plate reader. One-way ANOVA, GraphPad Prism, * *p* < 0.05, *** *p* < 0.005, **** *p* < 0.0001. (**b**) Representative images of immunofluorescence for lipid ROS after the staining with the C11 BODIPY 581/591 probe (on the left) and % of the mean fluorescence intensity of C11 BODIPY oxidized (green) vs. the mean fluorescence intensity of C11 BODIPY non-oxidized (red) calculated with ImageJ (on the right). One-way ANOVA, GraphPad Prism, * *p* < 0.05, **** *p* < 0.0001. (**c**) Representative images of immunofluorescence JC-1 staining (on the left) and % of the mean fluorescence intensity JC-1 aggregate (green) vs. the mean fluorescence intensity of JC-1 monomer (red) calculated with ImageJ (on the right). One-way ANOVA, GraphPad Prism, ** *p* < 0.01. (**d**) Representative results of a Seahorse XF Mito Stress Test Kit of A459 cells treated with different doses of NUs for 72 h. Plots on the right represent basal respiration and ATP production extracted from Mito Stress assay results obtained using the Seahorse Analyzer. Two-way ANOVA, GraphPad Prism. *** *p* = 0.0005, **** *p* < 0.0001.

To deepen the mechanism by which NUs induced cell stress and, ultimately, cell death in A549 cells, we investigated its pro-oxidant effects, given that AuNPs are known to act as oxidative agents, promoting lipid peroxidation, increasing ROS levels, and causing DNA damage and cell death [28]. Using higher concentrations of NUs, up to 24 μg/mL, which induced cell death within 72 h, we measured cellular oxidative stress using the CellROX reagent. A significant increase in ROS levels was observed following administration of 12 μg/mL NUs for 24 h, with ROS levels rising dramatically at 24 μg/mL (Figure 3a). To further confirm NUs-induced ROS enhancement, we assessed the oxidative degradation of cellular lipids using the BODIPY fluorescent probe. The results demonstrated pronounced pro-oxidant activity at 12 and 24 μg/mL after 24 h of treatment (Figure 3b).

Since mitochondria are central to ROS production [29], we investigated mitochondrial dysfunction using the JC-1 assay, an indicator of mitochondrial membrane potential (MMP). As shown in Figure 3c, after a brief exposure (3 h) to 24 μg/mL NUs, cells exhibited a marked shift in emission from red to green, reflecting MMP depolarization and decreased mitochondrial activity and integrity. Furthermore, considering the strong link between mitochondrial functionality and ATP production [30], we evaluated whether the early decrease in MMP observed after 3 h of NUs treatment could result in sustained mitochondrial dysfunction over longer exposure times. Using the Seahorse XF Mito Stress Test Kit, we found significant reductions in both basal respiration and ATP production after 72 h of treatment with 3 and 6 μg/mL NUs (Figure 3d). These findings suggest that NUs disrupt mitochondrial function, contributing to impaired energy metabolism and cell death.

### 3.3. Autophagy-Related Cell Death Induced by Nus in a459 Cells

**Figure 4:**
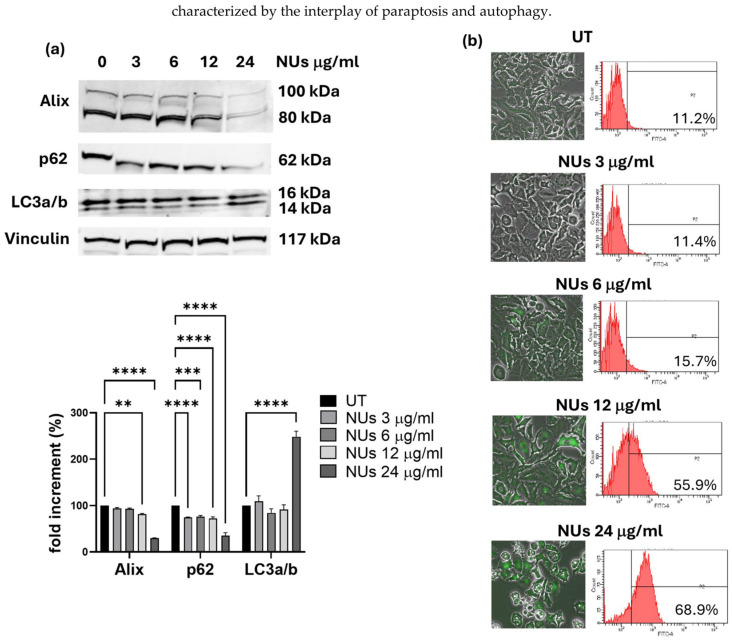
(**a**) Representative western blot analysis of Alix, p62, and LC3a/b and vinculin levels in A549 cells treated with different concentrations of NUs for 3 h. Two-way ANOVA, GraphPad Prism, ** *p* < 0.005, *** *p* = 0.0001, **** *p* = < 0.0001. (**b**) Fluorescent signals of autophagosomes stained with CYTO-ID^®^ dye detected using an inverted fluorescence microscope and quantified by FACS after 2 h of Nus treatment.

The extensive cytoplasmic vacuolation observed in A549 cells following NUs treatment prompted an investigation into the mechanisms of NUs-induced cell death. Cytoplasmic vacuolation is a hallmark of paraptosis, a caspase-independent form of programmed cell death [31]. To determine whether paraptosis was involved, we examined the expression of Alix, a known negative regulator of paraptosis. Western blot analysis revealed a significant downregulation of Alix expression after 3 h of NUs treatment, strongly suggesting that NUs induce paraptosis in A549 cells. Given the evidence in the literature linking paraptosis to autophagy in A549 cells [32], we further explored the potential involvement of autophagy in NUs-induced cell death. Western blot analysis of the autophagy markers p62 and LC3a/b demonstrated significant modulation of their levels after 3 h of NUs treatment, indicating the activation of autophagic processes alongside cell death (Figure 4a). 

To confirm autophagic involvement, we used the Enzo CYTO-ID autophagy detection kit to visualize autophagic vacuoles. Following 24 h of treatment with increasing concentrations of NUs, we observed a dose-dependent enhancement of green fluorescence, consistent with autophagic induction (Figure 4b). These findings suggest a mechanistic link between NUs-induced cytoplasmic vacuolation, paraptosis, and autophagy, with vacuole formation serving as a shared feature that bridges these processes. NUs appear to induce a unique form of cell death in A549 cells, characterized by the interplay of paraptosis and autophagy. 

### 3.4. NUs Biological Effects on A549 Cells

**Figure 5:**
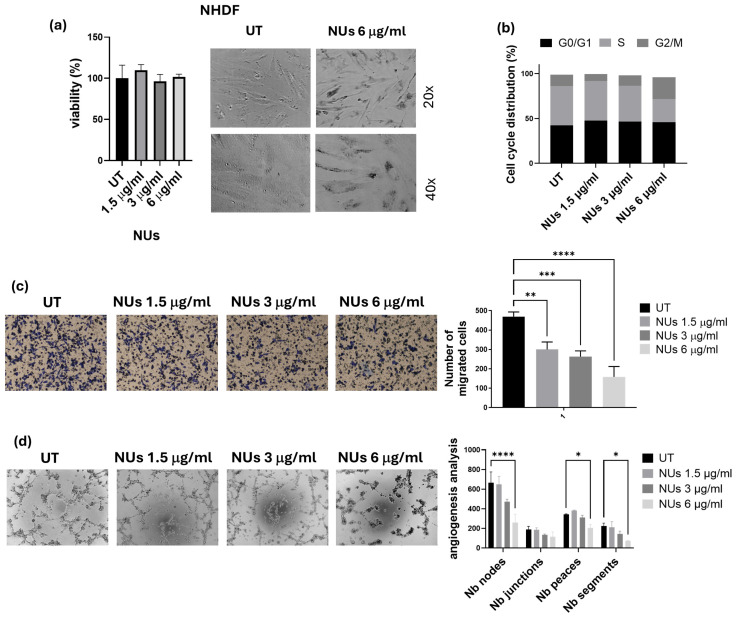
(**a**) MTT assay of NHDF cells treated with different doses of NUs for 72 h on the left and relative pictures obtained with an inverted microscope at 20× magnification after 6 μg/mL of NUs for 72 h on the right. (**b**) Cell cycle distribution analyzed by FACS after 24 h of treatment on A549 cells. (**c**) Representative images of invasiveness of A549 cells treated with different concentrations of NUs for 72 h (on the left) and quantitative analysis of the number of migrated cells through Geltrex on the right). One-way ANOVA, GraphPad Prism. ** *p* < 0.005, *** *p* < 0.0005, **** *p* < 0.0001. (**d**) Representative pictures (on the left) and relative quantification chart of capillary morphogenesis assay (on the right) of A549 treated with different concentrations of NUs for 72 h. Two-way ANOVA, GraphPad Prism. * *p* < 0.05, *** *p* < 0.005, **** *p* < 0.0001.

With the perspective of the potential clinical application of NUs, we first evaluated their biological effects on normal NHDF cells up to a concentration of 6 μg/mL, which was found to be non-toxic (Figure 5a). Given the safety profile of NUs for normal cells, we next focused on assessing the cellular damage in A549 cells.

Cell cycle analysis revealed a notable increase in the G2/M phase after 24 h of exposure to 6 μg/mL NU (Figure 5b), which aligned with the decrease in cell viability observed in the MTT assay. To determine the therapeutic potential of NUs in reducing malignant behaviors of tumor cells, we assessed their effects on cellular invasiveness and the ability of A549 to organize themselves in vascular-like structures, a process called vasculogenic mimicry (VM). NU treatment significantly inhibited cellular invasiveness after 6 h of incubation in A549 cells pre-treated for 72 h with 1.5, 3, and 6 μg/mL NU (Figure 5c). Furthermore, the highest dose (6 μg/mL) effectively reduced tube formation after 18 h of incubation, highlighting its potential to inhibit VM (Figure 5d). These findings suggest that NU not only exhibits non-toxic effects on normal cells but also has the potential to impair key tumor cell behaviors, such as invasiveness and vasculogenic mimicry, which are crucial for cancer progression.

### 3.5. NUs Potentiation of Cisplatin Toxicity on A459 Cells

**Figure 6:**
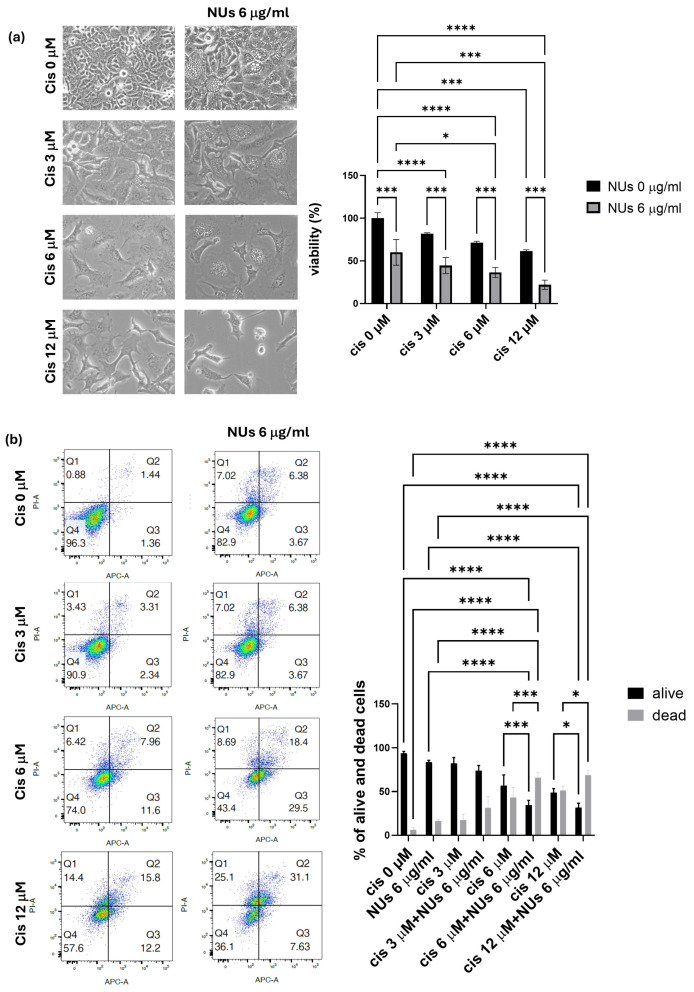
(**a**) MTT assay of A549 cells treated with different doses of cisplatin and NUs for 72 h. One-way analysis of variance (ANOVA), GraphPad Prism. * *p* < 0.05, *** *p* < 0.0005, **** *p* < 0.0001. (**b**) Cellular apoptosis of A549 cells after the treatment with different doses of cisplatin alone or in combination with NUs 6 μg/mL analyzed by FACS through cellular incorporation of PI and Annexin V-APC; a representative flow cytometry dot-plot picture on the left and relative quantification on the right. Two-way analysis of variance (ANOVA), GraphPad Prism * *p* < 0.05, *** *p* = 0.0001, **** *p* < 0.0001.

Based on the promising results obtained, we explored the potential of combining NUs with platinum-based chemotherapy, commonly used in the treatment of lung cancer, to enhance therapeutic efficacy. Various doses of cisplatin were tested alone or in combination with 6 μg/mL NUs, and the MTT assay revealed a significant synergistic effect between the two compounds after 72 h of incubation (Figure 6a).

Further analysis using PI and Annexin V staining, assessed by flow cytometry (FACS), confirmed that NUs potentiate cisplatin-induced cell death. These findings suggest that the combination treatment of NUs and cisplatin may offer a promising strategy for improving the effectiveness of platinum-based chemotherapy in lung cancer therapy.

## 4. Discussion

The application of nanotechnology in cancer has gained a great expansion in the past three decades [33,34], driven by the growing need to develop novel strategies to overcome drug resistance and improve targeted therapy, thereby minimizing the side effects associated with conventional chemotherapy. The unique properties of NPs, such as their size, surface area, and the ability to be functionalized, offer a versatile platform for enhancing drug delivery and targeting cancer cells with greater precision. In particular, metallic NPs, including gold, silver, and palladium, have garnered significant attention due to their stability, biocompatibility, and ability to facilitate cellular uptake [12]. Moreover, these particles demonstrate exceptional optical, catalytic, and antimicrobial properties, making them highly promising candidates for cancer therapy [11].

**Figure 7:**
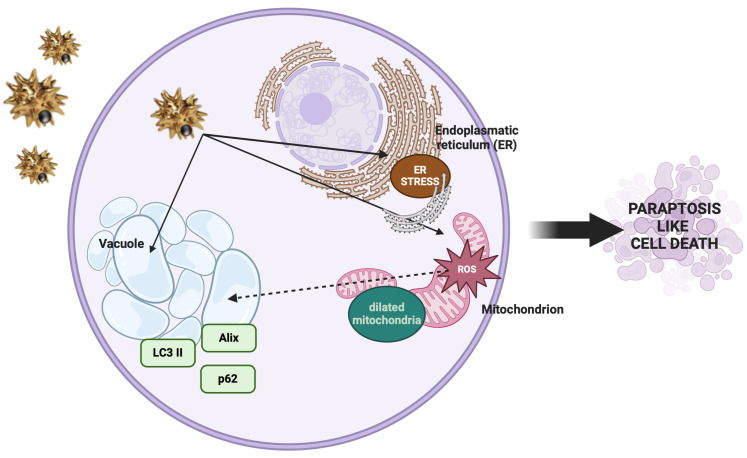
Cellular and molecular changes observed during paraptosis (created with BioRender).

In the present study, we investigated the anticancer potential of gold–iron oxide (Au-Fe_3_O_4_) NUs. Our findings demonstrate that NUs treatment induced significant cytotoxicity in melanoma (A375) and pancreatic (MIA PaCa-2) cancer cells, with MCF7 breast cancer cells also exhibiting considerable toxicity. In contrast, A549 lung cancer cells displayed a higher resistance to NUs treatment, though a substantial reduction in cell viability and cell death were observed at higher concentrations (24 μg/mL) after 72 h. These results highlight the varying sensitivity of cancer cell lines to NU treatment, suggesting that the response to the proposed NUs may be influenced by cell-specific factors such as membrane integrity, antioxidant capacity, and stress response pathways. Indeed, the higher resistance of A549 cells to NUs treatment appears to be primarily attributed to their elevated expression levels of antioxidant genes compared to the other tested cell lines, as shown in the Supplementary Materials (Supplementary Figure S1 and Table S1). This observation aligns with the existing literature, where A549 cells have been reported to produce fewer reactive oxygen species (ROS) than other cell lines, such as those from breast cancer or melanoma when treated with nanoparticles [35,36,37]. This reduced ROS production may contribute to the enhanced resistance of A549 cells to oxidative stress-induced cytotoxicity. One of the notable observations in MCF7 and more pronounced in A549 cells was the formation of cytoplasmic vacuoles following NUs treatment at sub-lethal doses. These vacuoles, which could be mistaken for lipid droplets, were negative for Oil Red O staining, indicating that they were not lipidic in nature. Instead, the vacuolization is consistent with cellular responses to stress and suggests that NU treatment may trigger a non-apoptotic form of programmed cell death. Indeed, the vacuoles observed in A549 cells bear characteristics of paraptosis, a caspase-independent form of cell death that is marked by the extensive dilation of the endoplasmic reticulum (ER) and mitochondria [38,39,40]. Oxidative stress is a key mediator of nanoparticle-induced cytotoxicity, particularly in cancer cells, where redox homeostasis is frequently altered. While tumor progression is often supported by elevated reactive oxygen species (ROS) production, cancer cells typically upregulate antioxidant defenses to maintain ROS levels within survivable limits [41]. The ability of metallic NPs to generate ROS is a promising strategy for bypassing this protective mechanism. In our study, treatment with NUs at concentrations of 12 and 24 μg/mL significantly increased ROS production and lipid peroxidation in A549 cells, leading to mitochondrial damage and eventual cell death after prolonged exposure. ROS generation is closely linked to mitochondrial dysfunction, which we confirmed by observing early depolarization of the mitochondrial membrane potential (MMP). This MMP depolarization, a hallmark of mitochondrial damage, was associated with a marked decrease in ATP production, as evidenced by Seahorse mitochondrial stress assays (Figure 3d). The sustained oxidative stress and mitochondrial dysfunction observed in A549 cells ultimately resulted in paraptosis, as characterized by cytoplasmic vacuolation and the absence of typical apoptotic markers such as membrane blebbing, chromatin condensation, or nuclear fragmentation [38,39,40]. Furthermore, we observed a significant reduction in the levels of Alix, a known endogenous inhibitor of paraptosis, following NUs treatment (Figure 4a), supporting the hypothesis that NUs-induced paraptosis is a key mechanism of cell death in A549 cells. This finding aligns with previous studies suggesting that ROS-induced paraptosis can be triggered by nanoparticles, with a growing body of evidence supporting the role of ROS generation in the initiation of this non-apoptotic death pathway [42,43,44]. Interestingly, the interplay between autophagy and paraptosis has been increasingly recognized as a critical factor in nanoparticle-induced cell death. Autophagy is a degradative process that can promote cell survival under stress conditions by recycling damaged cellular components, but when dysregulated, it can contribute to cell death [45,46]. In our study, we found that NUs treatment led to increased levels of LC3, a key marker of autophagy, and decreased levels of p62, suggesting that NUs promote autophagic flux (Figure 4a). The presence of autophagic vacuoles further supported this observation, indicating that the enhanced autophagic process was a precursor to paraptotic cell death. These findings highlight the complex relationship between autophagy and paraptosis in the context of nanoparticle-induced toxicity and suggest that targeting both pathways may enhance the therapeutic efficacy of NUs (Figure 7)

Our study also explored the potential use of lower NU doses that were non-toxic to normal human fibroblasts (NHDF cells), thereby suggesting that NUs may offer a selective approach to targeting cancer cells while sparing healthy tissue (Figure 5a). At sub-lethal concentrations, NUs treatment induced G2/M cell cycle arrest, a well-known consequence of ROS-induced DNA damage, further indicating the extent of cellular stress caused by NUs treatment in A459 (Figure 5b). In addition to inhibiting cell proliferation, NUs were shown to suppress the migratory and tube-forming capabilities of cancer cells, key behaviors associated with tumor progression and metastasis (Figure 5c,d).

Notably, NUs demonstrated a potentiation effect when used in combination with cisplatin, one of the most widely used chemotherapeutic agents [47]. Cisplatin resistance remains a major clinical challenge, and our findings suggest that NUs may enhance the sensitivity of cancer cells to cisplatin, thus offering a promising strategy to overcome chemoresistance in clinical settings. This synergy between NUs and cisplatin provides a strong rationale for future in vivo studies to evaluate the therapeutic potential of this combination in cancer treatment.

## 5. Conclusions

In conclusion, our study highlights the promising anticancer properties of Au-Fe_3_O_4_@PEG NUs, particularly in lung cancer cells, where NUs treatment induces sustained ROS production, mitochondrial dysfunction, and paraptosis-like cell death (Figure 6). The ability of NUs to potentiate cisplatin toxicity further underscores its therapeutic potential, and future investigations are warranted to explore its efficacy in preclinical and clinical models. Given the growing body of evidence supporting the role of metallic nanoparticles in cancer therapy, NUs represent a novel and valuable tool in the development of targeted cancer treatments.

It is important to note that the observed cytotoxic effects of these nanoparticles cannot be attributed solely to the metallic elements themselves, as demonstrated in the Supplementary Materials (Supplementary Figure S2). Our data show that gold nanorods and silver nanoparticles, when tested in lung cancer cells, do not induce the same level of toxicity as Au-Fe_3_O_4_@PEG NUs. This suggests that the cytotoxic effects of NUs are tissue-specific and may be related to the unique interactions of the nanoparticles with the lung cancer cells rather than the intrinsic properties of the metal alone.

## Data Availability

Data are contained within the article.

## Supplementary Materials

The following supporting information can be downloaded at: https://www.mdpi.com/article/10.3390/antiox14040422/s1, Figure S1: Real-Time PCR analyses for antioxidant enzymes in A549, MCF7, MIA-Paca-2 and A375M6 cells; Table S1: List of primers; Figure S2: MTT assay and pictures of A549 cells after different doses of gold nanoparticles (AuNRs) and silver nanoparticles (AgNPs) at different concentrations for 72 h. Reference [48] is cited in the Supplementary Materials.

## Abbreviations

The following abbreviations are used in this manuscript:

NUs	Nanourchins
NSCLC	Non-small-cell lung cancer
SCLC	Small-cell lung cancer
NPs	Nanoparticles
MMP	Mitochondrial membrane potential
ROS	Reactive oxygen species
