# Supplementary material for: Targeted Cancer Therapy with Gold–Iron Oxide Nanourchins: Inducing Oxidative Stress, Paraptosis, and Sensitizing Tumor Cells to Cisplatin
Source: Antioxidants (Basel). 2025 Mar 31;14(4):422. doi: 10.3390/antiox14040422 (PMC12024049; doi:10.3390/antiox14040422)
Supplement: Supplementary file 1 [file antioxidants-14-00422-s001.zip › antioxidants-3551559-supplementary.pdf]

Supplementary Materials:

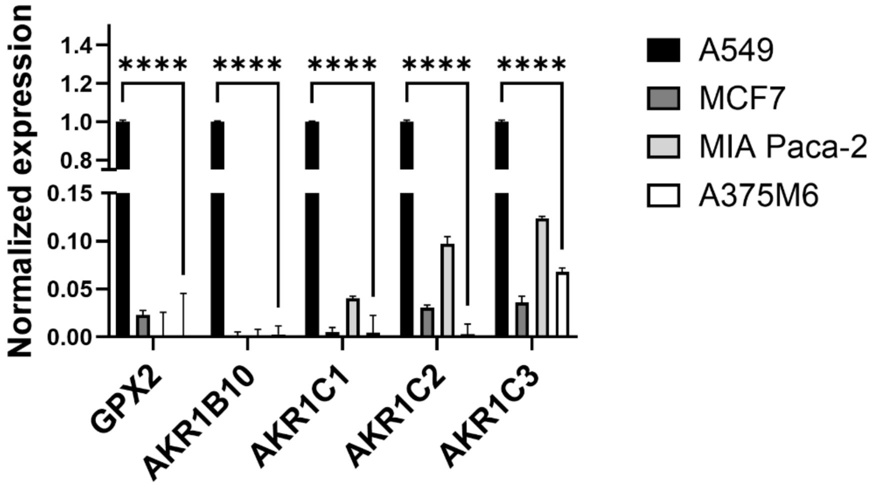

Figure S1. Real-Time PCR analyses for antioxidant enzymes in A549, MCF7, MIA-Paca-2 and A375M6 cells. Two-way ANOVA, GraphPad Prism, **** $p < 0.0001$ refers to A549 versus MCF7, MIA-Paca-2 and A375M6 cells for each analyzed gene.

Real-time PCR analysis revealed significantly higher expression levels of antioxidant enzymes in A549 cells compared to the other cell lines, which may account for their reduced sensitivity to NUs-induced cytotoxicity via the modulation of oxidative stress.

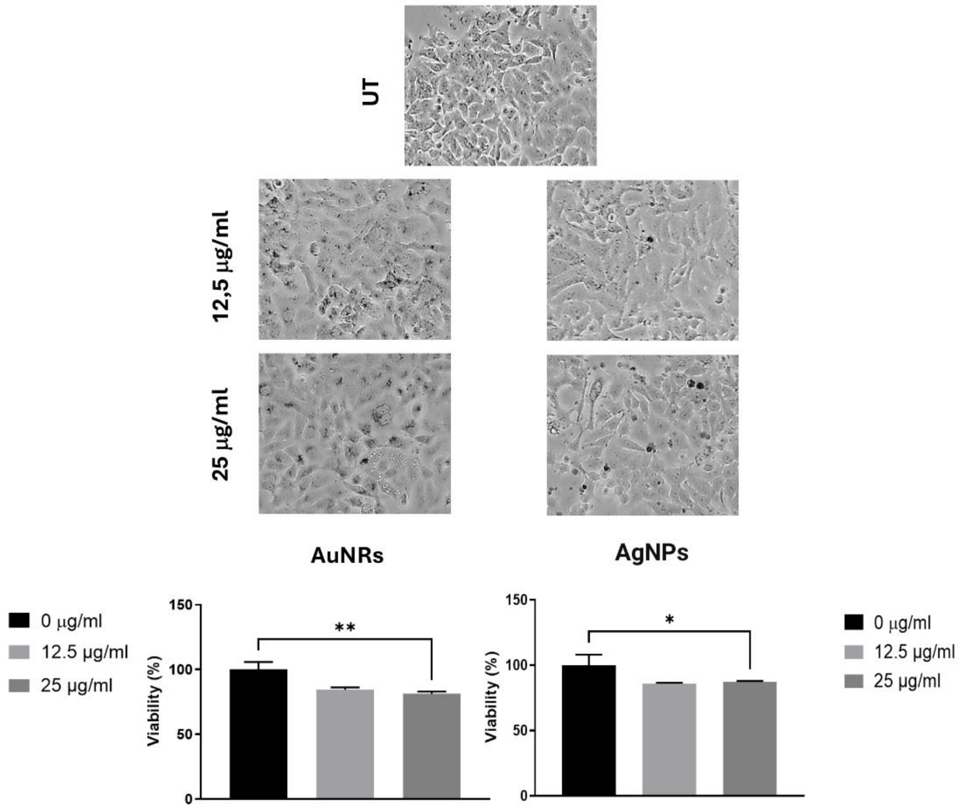

Figure S2. MTT assay and pictures of A549 cells after different doses of gold nanoparticles (AuNRs) and silver nanoparticles (AgNPs) at different concentrations for 72h. One –way ANOVA, GraphPad Prism, **p <0,01, **p<0,001*

The data indicate that, at the same administration time and dose, gold nanorods and silver nanoparticles exhibit lower cytotoxicity than NUs.

Supplementary materials and methods:

qRT-PCR:

As described by Peri et al. [1], A549 cells were lysed in TRI Reagent (Sigma Aldrich). Following the manufacturers' instructions, the RNA was extracted, solubilized in RNase/DNase free water and dosed with a NanoDrop™ One/OneCMicrovolume UV-Vis Spectrophotometer (Thermofisher, Monza, Italy). 1 µg of RNA was then retrotranscribed to the cDNA using the iScript™ cDNA Synthesis Kit (Biorad, Milan, Italy) and following the manufacturers' instructions. The cDNA was then diluted 1:5 in RNase/DNase free water. The real time PCR was performed in a CFX96 Touch Real-Time PCR Detection System (Biorad, Milan, Italy) using the primers listed below (Table 1). 1 µl 4 µM of forward and reverse primers mix, 2 µl of diluted cDNA, 2 µl of water and 5 µl of SsoAdvanced Universal SYBR Green Supermix were dispensed into a 96 well plate with each experimental point in triplicate. Real time was performed with the following steps: (1) 2 min at 95° C, (2) 15 s at 95° C, (3) 30 s at 60° C, (4) repeat from step 2, 39 more times (5) and then increase the temperature from 55° C to 95° C, increasing 0.5 C/s. The data were then analyzed using the CFX Maestro software (Biorad, Milan, Italy).

Target Gene	Forward	Reverse
18S	5′-CGGCTACCACATCCAAGGAA-3′	5′-GCTGGAATTACCCGCGCT-3′
GPX2	5′-CCCTTGCAACCAATTTGGAC-3′	5′-TCCTTCAGGTAGGCGAAGAC-3′
AKRIB10	5′-CCAAGTCTGTGACACCAGCA-3′	5′-CGTTACAGGCCTCCAGTTT-3′
AKRIC1	5′-TGCATAATGCCTGGGCTATCTT-3′	5′-AGGCCATGACAGTGTTTGAG-3′
AKRIC2	5′-GACCAGCCTTGGAAAGGTCA-3′	5′-AGACATGCAATCACGGAAGT-3′
AKRIC3	5′-ATGCCTGTCCTGGGATTTGG-3′	5′-GGCGGAACCCAGCTTCTATT-3′

Table S1. List of primers

Gold nanorods (AuNRs) were synthesized by Prof. Fulvio Ratto at the Institute of Applied Physics, National Research Council. These nanorods, exhibiting a longitudinal plasmonic oscillation mode in the first biological window, were synthesized and stabilized with cetyltrimethylammonium bromide (CTAB), following the method reported by X. Ye et al. The plasmonic band of the nanorods peaks around 810 nm**.** [2].

Silver nanoparticles (AgNPs) were synthesized by Prof. Eleni K. Efthimiadou at the Department of Chemistry National and Kapodistrian, University of Athens. 1000 mg of tannic acid and 100 mg of NaOH were added in 25 ml of H_2O and the suspension was magnetically mixed for 10 minutes. 2,5 ml of H_2O and 500 mg of $AgNO_3$ were added and after mixing for 5 minutes at room temperature, further 25 ml of H_2O were added. The obtained solution was centrifuged, washed with double distilled water and centrifuged again. Finally Ag content was measured.

References

1. Peri, S.; Ruzzolini, J.; Urciuoli, S.; Versienti, G.; Biagioni, A.; Andreucci, E.; Peppicelli, S.; Bianchini, F.; Bottari, A.; Calorini, L.; et al. An Oleocanthal-Enriched EVO Oil Extract Induces the ROS Production in Gastric Cancer Cells and Potentiates the Effect of Chemotherapy. *Antioxidants* **2022**, *11*, 1762, doi:10.3390/antiox11091762.
2. Ye, X.; Zheng, C.; Chen, J.; Gao, Y.; Murray, C.B. Using Binary Surfactant Mixtures To Simultaneously Improve the Dimensional Tunability and Monodispersity in the Seeded Growth of Gold Nanorods. *Nano Lett.* **2013**, *13*, 765–771, doi:10.1021/nl304478h.
